# Immune cell infiltration signatures identified molecular subtypes and underlying mechanisms in gastric cancer

**DOI:** 10.1038/s41525-021-00249-x

**Published:** 2021-10-11

**Authors:** Yilin Lin, Xiaoxian Pan, Long Zhao, Changjiang Yang, Zhen Zhang, Bo Wang, Zhidong Gao, Kewei Jiang, Yingjiang Ye, Shan Wang, Zhanlong Shen

**Affiliations:** 1 Department of Gastroenterological Surgery, Peking University People’s Hospital, Beijing, 100044 PR China; 2 Laboratory of Surgical Oncology, Beijing Key Laboratory of Colorectal Cancer Diagnosis and Treatment Research, Peking University People’s Hospital, Beijing, 100044 PR China; 3 Department of Radiotherapy, First Affiliated Hospital of Fujian Medical University, Fuzhou, Fujian 350004 PR China

**Keywords:** Cancer microenvironment, Cancer immunotherapy, Data mining

## Abstract

Increasing evidence has clarified that the tumor microenvironment (TME) is closely related to the prognosis and therapeutic efficacy of cancer. However, there is no reliable TME evaluation system used to accurately predict the prognosis of and therapeutic efficacy in gastric cancer. We evaluated the immune microenvironment score (IMS) of 1422 gastric cancer samples based on 51 immune cell signatures. We explored the relationship between the IMS and prognosis, immune cell infiltration, cancer subtype, and potential immune escape mechanisms. The results show that activation of the stroma and decreased levels of immune infiltration were associated with a low IMS. A high IMS was characterized by Epstein–Barr virus infection, increased mutation load, microsatellite instability, and immune cell infiltration. A high IMS was also related to high expression of immune checkpoint molecules (PD-1/PD-L1). Finally, patients with a high IMS had a better response to PD-1/PD-L1 inhibitors and may be more suitable for immune checkpoint inhibitors (area under the curve = 0.81). In addition, a low IMS may be converted into the immune-infiltrating subtype after romidepsin treatment. Stratification based on the IMS may enable gastric cancer patients to benefit more from immunotherapy and help identify new cancer treatment strategies.

## Introduction

Gastric cancer is the fifth most common malignant tumor and the fourth leading cause of cancer death^1^. Although its morbidity and mortality have declined in the past few years, gastric cancer is still a serious global health problem^2–4^. At present, surgery, chemotherapy, radiation therapy, and targeted therapy are the main treatment methods for gastric cancer^5^. The American Joint Committee on Cancer (AJCC) staging system and histological classification are the most important tools for the stratification, classification, and treatment of patients with gastric cancer^6,7^. In recent years, high heterogeneity has been found in gastric cancer, and new stratifications have been proposed for gastric cancer^8^. In addition, it is necessary to identify other important factors to stratify patients more precisely with gastric cancer to better guide clinical treatment and improve prognosis.

Immunotherapy has emerged as a treatment option in recent years and mainly induces antitumor effects by regulating the immune system, and immunotherapy has achieved revolutionary progress in the treatment of malignant tumors^9^. However, the current dilemma facing immunotherapy is the lack of accurate prediction of efficacy. The tumor microenvironment (TME) is a complex ecosystem consisting of various types of cells and other noncellular components of the extracellular matrix with obvious heterogeneity^10^. Recent studies have provided an in-depth understanding of the TME, which has provided new opportunities for immunotherapy^11–13^. Studies suggest that a positive response to immunotherapy usually depends on the dynamic interaction between tumor cells and immunomodulators in the TME^14–16^. Tumor cells inhibit the response and function of infiltrating immune cells by affecting the PD-1/PD-L1 signaling pathway and secreting inhibitory factors such as interleukin 2 (IL-2) to induce immune escape^17^. The TME recruits and expands immunosuppressive cells, such as regulatory T cells (Tregs), tumor-associated macrophages, and bone marrow-derived suppressor cells, which are some of the main effector cells inducing an immunosuppressive TME^18–20^. One study divided the TME into different phenotypes based on the degree of immune cell infiltration: immunoinflammatory phenotype, exclusion phenotype, and desert phenotype; this study also proposed the importance of selecting appropriate treatment strategies according to the phenotype of effector immune cells^21^. Cancer-associated fibroblasts, which are present in large numbers, in the TME form a high-density extracellular matrix, which hinders the absorption of drugs and the intratumoral infiltration of immune cells, leading to different immune responses^22^. Studies have revealed that the TME has high heterogeneity and an influence on the immune response. Therefore, the development of more precise stratification methods considering the state of the TME is urgent for optimizing the efficacy of immunotherapy.

At present, some researchers have analyzed the effect of the heterogeneity of the TME on immunotherapy. Peng et al. constructed a signature of T cell dysfunction and exclusion, and it was found that this signature was more accurate than PD-L1 expression and tumor mutation burden (TMB) in predicting the efficacy of immunotherapy in melanoma^23^. Yang et al. also constructed an immune-related signature to predict the efficacy of immunotherapy^24^. In addition, Zeng et al. evaluated the immune subtypes of gastric cancer through the CIBERSORT algorithm, thereby establishing the TME score of gastric cancer patients to evaluate the patient’s immune efficacy^25^. These studies have revealed the importance of the TME score in immunotherapy, but their research is only for the analysis of T cells or stratification according to the CIBERSORT algorithm. The CIBERSORT algorithm only counts 22 TME cells, and most TME cells were not included^26^. Therefore, it is urgent to adopt a new evaluation system to include more TME cells for stratified analysis.

In this study, we focused on evaluating TME characteristics to study the immune activity, prognosis, and immunotherapy response in gastric cancer. Fifty-one TME cells were included, and 27 TME cell types associated with the survival of gastric cancer were identified based on six independent cohorts of patients with gastric cancer. Based on these 27 TME cell types, an immune microenvironment score (IMS) for gastric cancer was established. The relationship of the IMS with gene expression profiles, somatic copy number variations (SCNVs), and mutations was analyzed. We found that the IMS is a powerful prognostic biomarker and predicts the response to immune checkpoint inhibitors. The flowchart of this research is shown in Supplementary Fig. 1

## Results

### Identification of survival-related immune cells used to construct IMS

**Fig. 1:**
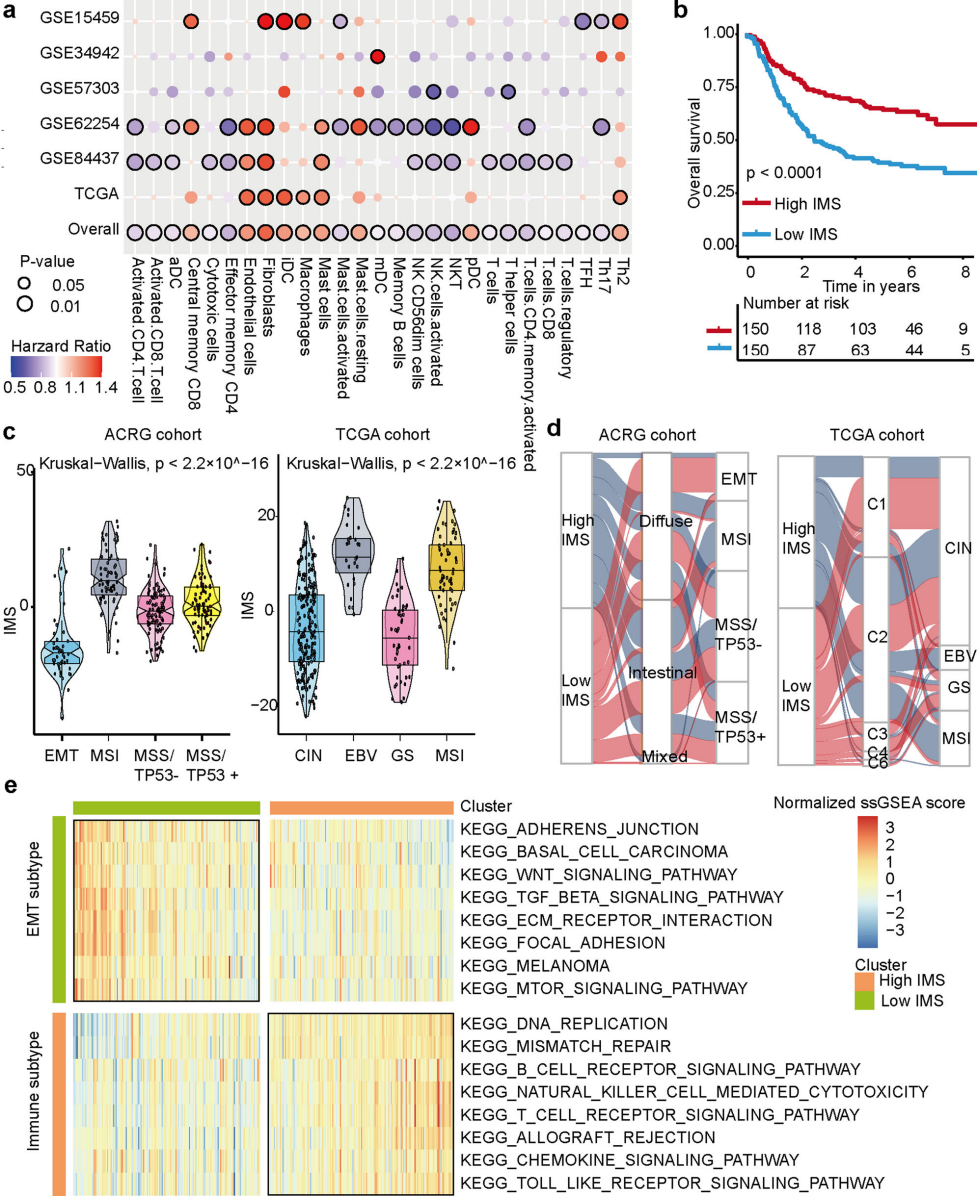
The distribution of the IMS in gastric cancer. **a** Immune cell types that have survival significance in gastric cancer. A meta-analysis was used to evaluate the overall survival effect of each immune cell type. Circles with a black border represent survival significance in an independent cohort as determined through univariate Cox analysis. Red points indicate an HR value >1, implying a potential risk factor. Blue points indicate an HR value less than 1, implying a potential favorable factor. The size of the circle represents the level of the HR. **b** Kaplan–Meier curve of OS according to the IMS in the ACRG cohort (log-rank test, *P* value < 0.0001). **c** The relationship between gastric cancer subtypes and the IMS (Kruskal–Wallis, *P* value < 2.2 × 10^–16^). The central line represents the median value. The bottom and top of the boxes are the 25th and 75th percentiles (interquartile range). The whiskers encompass 1.5 times the interquartile range. **d** Sankey diagram showing the relationship between the IMS and gastric cancer subtypes. **e** KEGG pathways enriched in the high and low IMS groups were analyzed by GSVA. Heatmaps were used to visualize these signaling pathways. *P < 0.05, **P < 0.01, ***P < 0.001, ****P < 0.0001.

To develop an IMS for gastric cancer, six gastric cancer cohorts were included (Supplementary Data 1). A total of 51 TME cell signatures from previously published research were analyzed. Single-sample gene set enrichment analysis (ssGSEA) was used to estimate the immune enrichment score based on each of the 51 TME cell signatures for each gastric cancer patient in each cohort. Univariate Cox regression analysis was used to estimate the utility of the immune enrichment score for survival in each gastric cancer cohort. The results indicated that a total of 38 TME cells were associated with the survival of gastric cancer (Supplementary Data 2). Meta-analysis revealed that a total of 27 TME cells were significantly related to the survival of gastric cancer in the overall cohort (*P* values < 0.05, Fig. 1a and Supplementary Data 3). The infiltration characteristics of these 27 TME cells are shown by the heatmap in the ACRG and The Cancer Genome Atlas (TCGA) cohorts (Supplementary Fig. 2). A total of 27 survival-related TME cells containing 463 marker genes are shown in Supplementary Data 4.

Next, we calculated the IMS of each gastric cancer patient in the cohort and divided the patients into a high IMS group and a low IMS group by using the median IMS as the cutoff value. The results revealed that patients with a high IMS had a longer survival time than patients with a low IMS in the ACRG cohort (*P* values < 0.05, Fig. 1b).

### The relationship between the IMS and clinical features in gastric cancer

Gastric cancer was divided into four subtypes in the ACRG cohort. Our research found that the IMS was the highest in the microsatellite instability (MSI) subtype, followed by the MSS/TP53-negative subtype and the MSS/TP53-positive subtype. The IMS level was the lowest in the epithelial-mesenchymal transition (EMT) subtype (Fig. 1c). There were significant differences in the IMS between the four subtypes (Kruskal–Wallis test, *P* value < 2.2e–16). Similarly, we also found that the IMS was the highest in the Epstein–Barr virus (EBV) subtype and MSI subtype in the TCGA cohort (Fig. 1c, Kruskal–Wallis test, *P* value < 2.2e–16).

We further explored the relationship between IMS and other clinical features. We found that patients with gastric cancer aged >65 years had higher IMSs than those aged ≤65 years in the ACRG and TCGA cohorts (*P* values < 0.05, Supplementary Fig. 3a). There was no significant difference in IMS between males and females in the ACRG cohort (*P* value = 0.093, Supplementary Fig. 3b). However, females had higher IMSs than males in the TCGA cohort (*P* value = 0.035). Next, we continued to analyze the relationship between the IMS and the AJCC stage. The results showed that the IMS was the highest in stage I disease, while the IMS was the lowest in stage III and stage IV disease (*P* value = 2.04 e−17, Supplementary Fig. 3c), indicating that the IMS may have been related to the progression of gastric cancer in the ACRG cohort. However, in the TCGA cohort, although the IMS also showed the highest expression in stage I disease, there was no significant difference in the IMS between disease stages in this cohort (*P* value = 0.38). Therefore, more data and meta-analyses may be needed to obtain a more reliable conclusion. Next, we further analyzed the pathological subtypes (the intestinal type, diffuse type, and mixed type) in the ACRG cohort. Since the sample size of mixed subtypes is small (eight samples), we exclude these samples here. The IMS of intestinal-type patients was significantly higher than that of diffuse-type patients (all *P* values < 0.05, Supplementary Fig. 3d). In addition, MLH1-negative gastric cancer had a higher IMS than MLH1-positive gastric cancer (Supplementary Fig. 3d). Gastric cancer was also divided into six subtypes in the TCGA cohort. The six subtypes were C1 (wound healing), C2 (IFN-γ dominant), C3 (inflammatory), C4 (lymphocyte depleted), C5 (immunologically quiet), and C6 (TGF-β dominant). Our results showed that C2 samples had a high IMS (Supplementary Fig. 3e). A Sankey diagram of these data was generated with the R package ggalluvial (Supplementary Data 5 and Fig. 1d).

To further analyze the characteristics of the high and low IMS groups, KEGG functional enrichment analysis of the gastric cancer samples was performed with ssGSEA. To our surprise, the low IMS group was highly enriched in stromal and oncogenic pathways, such as the mTOR signaling pathway, WNT signaling pathway, adherens junction pathway, TGF-beta signaling pathway, and focal adhesion pathway. The high IMS group was enriched in pathways related to dMMR and immune activation, including mismatch repair, natural killer cell-mediated cytotoxicity, Toll-like receptor signaling, T cell receptor signaling, B cell receptor signaling, and chemokine signaling pathways (Fig. 1e).

### Landscape of IMS in gastric cancer

**Fig. 2:**
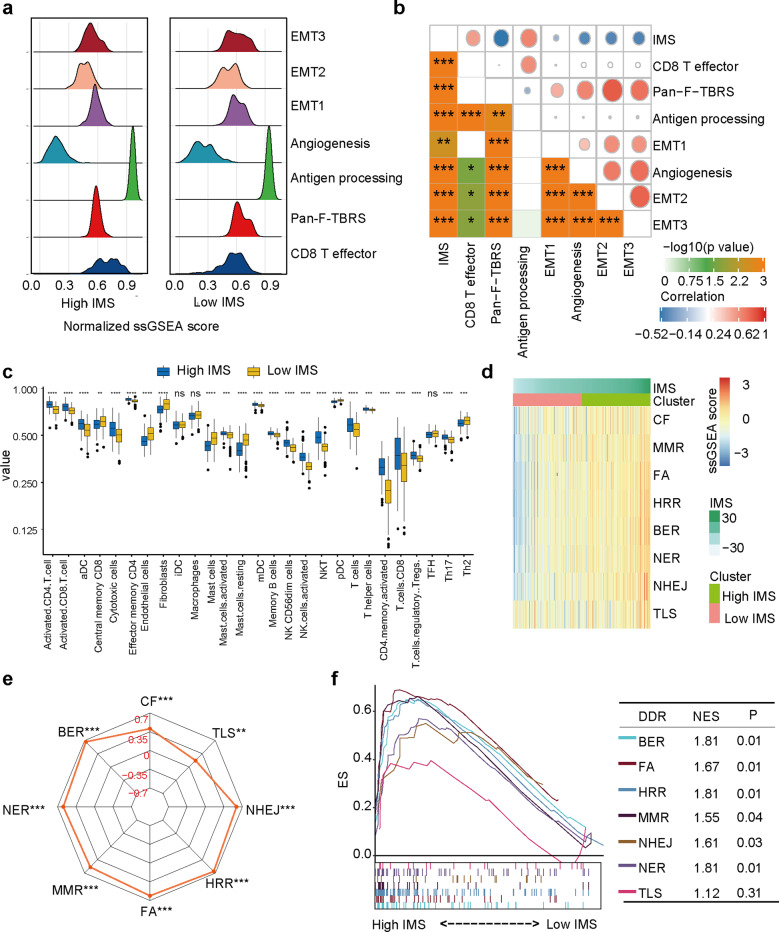
The effect of the IMS in gastric cancer (ACRG cohort). **a** Differences in immune-related pathways and stroma-activated pathways, including the EMT, TGF-beta, angiogenesis, effector CD8 T cell, and antigen presentation pathways, between the high and low IMS groups (Wilcoxon test). **b** The relationship between the signature scores of immune-related pathways and stroma-activated pathways and the IMS (Spearman analysis). **c** The differential expression of 27 survival-related immune cell types in patients with high and low IMS. The central line represents the median value. The bottom and top of the boxes are the 25th and 75th percentiles (interquartile range). The whiskers encompass 1.5 times the interquartile range. **d** Heatmap showing the enrichment scores of eight DNA damage repair (DDR) pathways in gastric cancer samples. **e** Correlation analysis between the enrichment scores of the eight DNA pathways and the IMS (Spearman analysis). **f** GSEA of DDR pathways for the high IMS group versus the low IMS group. *P < 0.05, **P < 0.01, ***P < 0.001, ****P < 0.0001.

We assessed a set of genes related to specific biological processes identified by Mariathasan et al.^27^. In this analysis, high expression of EMT markers, including EMT1, EMT2, and EMT3, angiogenesis characteristics, and panfibroblast TGFβ response characteristics (Pan-F TBRS) were also found in the low IMS group. The CD8 effector and antigen presentation signatures were significantly highly expressed in the high IMS group (Fig. 2a). The Spearman correlation analysis results confirmed that these signatures were significantly related to the IMS (Fig. 2b).

Next, we analyzed the infiltration level of 27 cells in high and low IMS. The results showed that most cells had significant differences between the high-IMS and low IMS groups (Fig. 2c).

Our previous research found that a high IMS was related to MSI (Fig. 1c, e). For this reason, we were interested in the relationship between IMS and DNA damage repair pathways. We analyzed a total of eight DNA damage repair signatures (CF, MMR, FA, HRR, BER, NER, NHEJ, and TLS). We were surprised to find that these pathways were significantly differentially enriched between the high and low IMS groups (Fig. 2d), and the IMS was significantly positively correlated with the normalized enrichment scores (NESs) of these pathways (Fig. 2e). In addition, most of the results were verified with GSEA software (Fig. 2f).

### Potential extrinsic immune escape mechanism in gastric cancer (TCGA cohort)

Previously published studies have explained that different tumor subtypes may have different extrinsic immune escape mechanisms, such as suppression of immune cells, activation of immunosuppressive cells, and high expression of immunosuppressive cytokines^28^. Our previous study found that the low IMS group had more quiescent cells and activated innate immune cells (Treg cells, resting mast cells, and plasmacytoid dendritic cells (pDCs)) than the high IMS group, suggesting that patients in the low IMS group may have an insufficient adaptive immune response (Fig. 2c). The high IMS group had not only abundant and active innate and adaptive immune cells but also immunosuppressive cells (Treg cells) (Fig. 2c), suggesting that immunosuppressive cells may play a role in immune escape in this subtype. Our further analysis found that both high and low IMS samples had higher expression of chemokines, ILs, and interferon than adjacent normal tissue samples (Supplementary Fig. 4a). Moreover, the high IMS group had higher expression of immunostimulatory and immunosuppressive cytokines than the low IMS group. We wanted to know whether this difference was caused by SCNV, but the results showed no significant differences in SCNV between the high and low IMS groups (Supplementary Fig. 4b).

### Potential intrinsic immune escape mechanisms in gastric cancer (TCGA cohort)

**Fig. 3:**
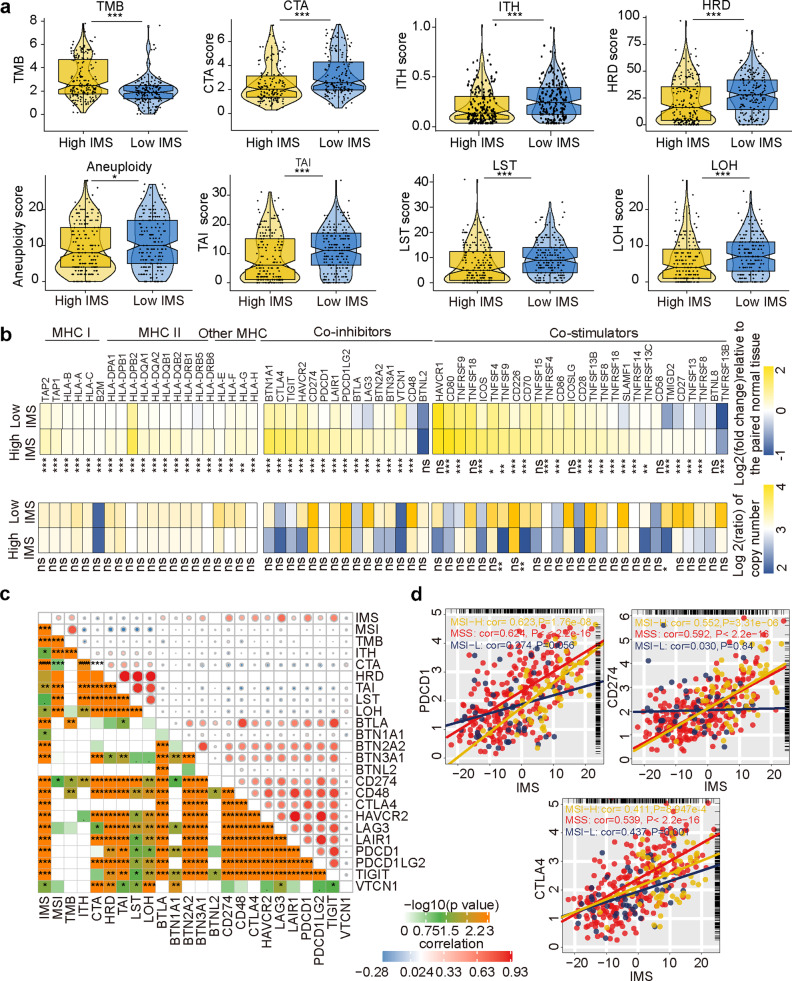
Correlation between the IMS and potential intrinsic immune escape mechanisms in gastric cancer (TCGA cohort). **a** Top: comparison of tumor mutation burden (TMB), cancer testis antigen (CTA) score, intratumoral heterogeneity (ITH) score, and homogeneous recombination deficiency (HRD) score between the high and low IMS groups. Bottom: comparison of aneuploidy score, loss of heterozygosity (LOH) score, telomeric allelic imbalance (TAI) score, and large-scale state transition (LST) score between the high and low IMS groups. The central line represents the median value. The bottom and top of the boxes are the 25th and 75th percentiles (interquartile range). The whiskers encompass 1.5 times the interquartile range. **b** Top: comparison of the log2-fold changes in MHC, costimulator, and coinhibitor mRNA expression at the tumor site relative to that in paired normal tissue. Bottom: comparison of the log2 ratio of the copy number values of MHC molecules, costimulators, and coinhibitors for the high and low IMS groups. **c** Correlation between the IMS and immune checkpoint molecule expression and the tumor immunogenicity score. **d** Scatter plot revealing the correlation between the IMS and immune checkpoint molecule (CD274, PDCD1, and CTLA4) expression in different subtypes (MSS, MSI-S, and MSI-H). *P < 0.05, **P < 0.01, ***P < 0.001, ****P < 0.0001, ns *P* value > 0.05.

We analyzed some factors related to tumor immunogenicity, such as TMB, cancer testis antigen (CTA), IHT, homologous recombination repair deficiency (HRD) (telomeric allelic imbalance (TAI), large-scale state transition (LST), and loss of heterozygosity (LOH)), chromosomal aneuploidy, and antigen-producing ability (Fig. 3a). The high IMS group had a higher TMB (*P* value < 0.05), and the low IMS group had higher levels of CTA, IHT, HRD (including TAI, LST, and LOH), and chromosomal aneuploidy. However, we further compared the two groups and found that the high IMS group had higher expression of MHC I-related antigen-presenting molecules than the low IMS group (all *P* < 0.001, Fig. 3b, left). We employed a list of costimulatory and coinhibitory molecules (https://www.rndsystems.com/cn/research-area/co-stimulatory-and-co-inhibitory-molecules) to compare the expression of immunomodulatory molecules between the high and low IMS groups. The results showed that the high IMS group had higher expression of immunomodulatory molecules than the low IMS group (most *P* values < 0.05, Fig. 3b, right). However, SCNV cannot explain the difference in the expression of molecules between the two groups (most *P* > 0.05, Fig. 3b).

In addition, we proved that there was a significant positive correlation between the IMS and MSI, TMB, and immune checkpoint molecule expression, suggesting a correlation between immunogenicity and immune checkpoint molecules. The IMS and other immunogenicity indicators (the CTA, HRD, and intratumoral heterogeneity (ITH) scores) were significantly negatively correlated (Fig. 3c). However, the expression of immune checkpoint molecules was not significantly correlated with immunogenicity indicators (Fig. 3c). Finally, we further analyzed the correlation between the IMS and the expression of immune checkpoint molecules (PDCD1, CD274, and CTLA4) in different subtypes based on microsatellite stability status (MSS, MSI-L, and MSI-H) (Fig. 3d). Our results revealed that the IMS was significantly positively correlated with the expression of the three immune checkpoint molecules in the MSS and MSI-H subtypes (all *P* values < 0.05). However, the IMS was not significantly related to the expression of PDCD1 and CD274 in the MSI-L subtype (*P* value > 0.05).

### Correlation of genomic alterations with the IMS in gastric cancer (TCGA cohort)

**Fig. 4:**
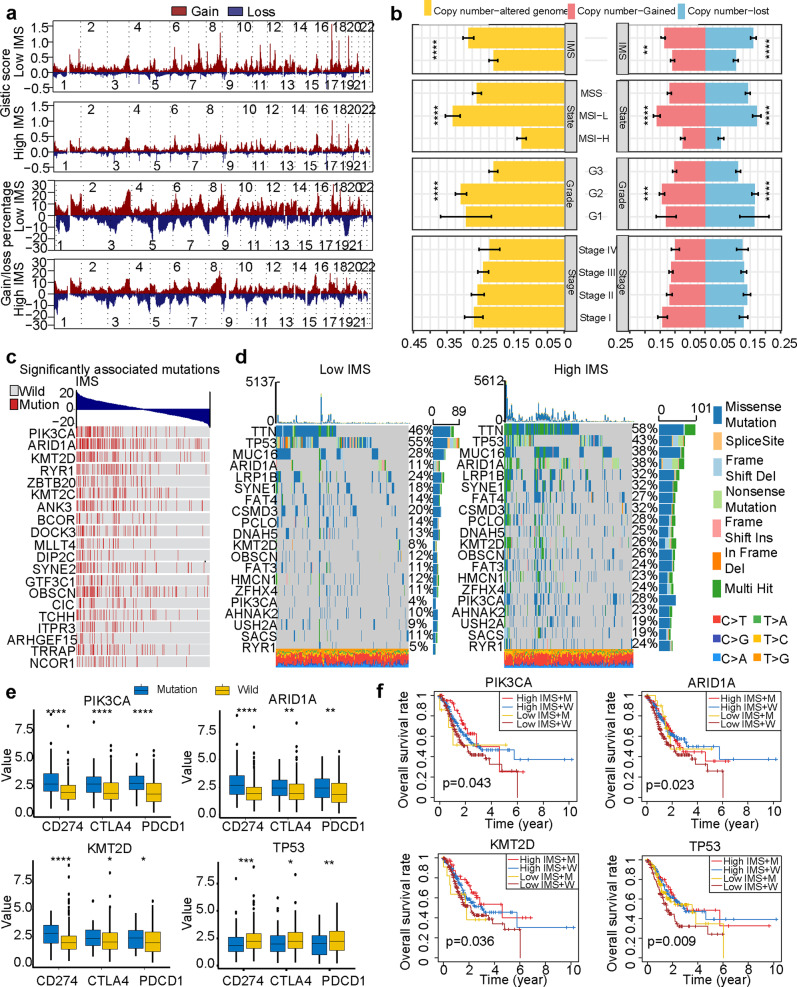
Correlation of genomic alterations with the IMS in gastric cancer. **a** Comparison of the somatic copy number variations (SCNVs) between the high IMS group and the low IMS group in the TCGA gastric cancer cohort. **b** Differences in fraction genome altered (FGA), fraction genome gain (FGG), and fraction genome loss (FGL) values in different AJCC stages, pathological subtypes, and IMS subtypes (Kruskal–Wallis). **c** Heatmap showing mutations in the genes of interest in gastric cancer samples. These mutations were significantly related to the IMS in the TCGA gastric cancer cohort. **d** Top 20 most frequently mutated genes associated with the IMS in the TCGA gastric cancer cohort. **e** PIK3CA, ARID1A, and KMT2D mutations significantly increased the expression of immune checkpoint molecules (CD274, PDCD1, and CTLA4). TP53 mutation significantly reduced the expression of immune checkpoint molecules (CD274, PDCD1, and CTLA4). The central line represents the median value. The bottom and top of the boxes are the 25th and 75th percentiles (interquartile range). The whiskers encompass 1.5 times the interquartile range. **f** The relationship between mutations in PIK3CA, ARID1A, KMT2D, and TP53 and prognosis. *P < 0.05, **P < 0.01, ***P < 0.001, ****P < 0.0001.

We further explored the relationship between the IMS and genome alterations (including SCNVs and mutations). We first analyzed the GISTIC scores and copy number gain/loss frequencies in the high and low IMS subtypes. The results showed that the low IMS group had higher GISTIC scores and copy number gain/loss frequencies than the high IMS group. The low IMS group also had a higher copy number gain/loss percentage (Fig. 4a). Then, we analyzed the differences in fraction genome altered (FGA), fraction genome gain (FGG), and fraction genome loss (FGL) among different subtypes. We found that there was no significant difference between the different AJCC stages (Fig. 4b). However, the MSI-L subtype had the highest FGA, FGG, and FGL values, while the MSI-H subtype had the lowest FGA, FGG, and FGL values (Fig. 4b). This result suggests that an increased copy number gain/loss frequency may be a factor contributing to a low IMS in gastric cancer patients.

Next, we analyzed the association between gene mutations and the IMS. The mutation status of 4714 genes was significantly correlated with the IMS by Spearman’s analysis (|Spearman’s correlation| > 0.1, *P* value < 0.05, Supplementary Data 6). Based on the correlation coefficient, the top 20 gene mutations that were significantly related to the IMS are illustrated in Fig. 4c. Among the 4714 genes, we included the top 20 genes with the highest mutation frequency in the total TCGA cohort in Fig. 4d. We found that PIK3CA, ARID1A, and KMT2D were among the 20 most frequently mutated genes and were involved in the top 20 mutations that were significantly related to the IMS. Moreover, most of the gene mutation frequencies in the high IMS group were higher than those in the low IMS group, except for the TP53 gene mutation frequency. Therefore, we wanted to explore whether these gene mutations were a potential intrinsic immune escape mechanism of the high and low groups. The results showed that the expression of immune checkpoint molecules (CD274, PDCD1, and CTLA4) in the groups with PIK3CA, ARID1A, and KMT2D mutations was mostly significantly higher than that in the wild-type group (Fig. 4e), and the expression of immune checkpoint molecules in the TP53-mutated group was significantly lower than that in the wild-type group. Finally, we analyzed whether these genes affect the survival of patients with high IMS and low IMS. Although the overall *P* value was significant, mutations in these four genes did not affect the survival of patients with high IMS or low IMS (Fig. 4f).

### IMS is a marker for prognosis and can predict postoperative adjuvant treatment benefits

**Fig. 5:**
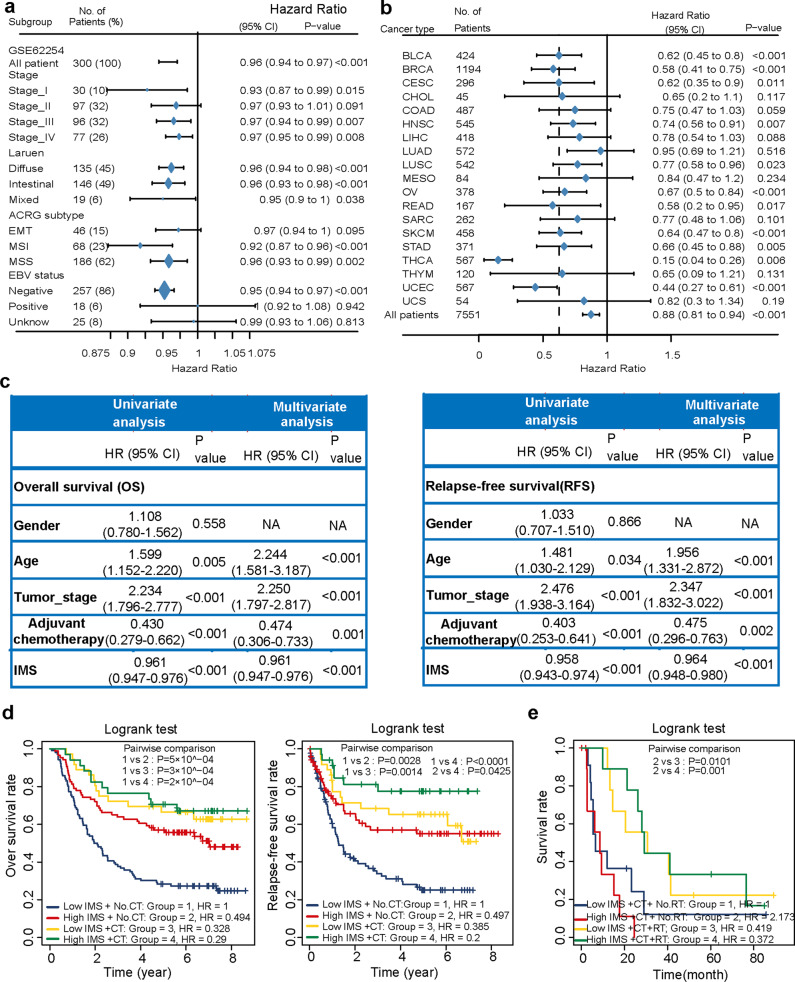
Prediction of gastric cancer prognosis and postoperative adjuvant treatment response. **a** Univariate Cox regression was used to analyze the prognostic value of the IMS in subgroups based on subtype or clinical characteristics. An HR < 1.0 indicates that a high IMS is a favorable prognostic factor. **b** Univariate Cox regression analysis revealed the IMS as a favorable prognostic factor for 19 cancers. A meta-analysis was performed to evaluate the prognostic value of the IMS for pancancer. **c** Univariate and multivariate Cox regression analysis of the relationship between sex, age, postoperative chemotherapy, and IMS and prognosis. The results show that age, postoperative adjuvant chemotherapy, and IMS are independent prognostic factors. **d** The results showed that postoperative chemotherapy can reduce the risk of recurrence and prolong the survival of patients with high and low IMS. CT chemotherapy. The log-rank test was used to perform survival analysis, and significant differences are displayed in the figure. **e** Postoperative radiotherapy can significantly improve the prognosis of patients with high and low IMS. The log-rank test was used to perform survival analysis, and significant differences are displayed in the figure. RT radiotherapy.

We stratified gastric cancer samples to estimate the relationship of IMS with overall survival in the ACRG cohort (Fig. 5a). Significant differences were observed among most subtypes. We also analyzed the survival of the high and low IMS groups in the other four cohorts (Supplementary Fig. 5a). The meta-analysis results showed that a high IMS was associated with a longer survival time than a low IMS in gastric cancer (Supplementary Fig. 5b). To explore whether the IMS had a strong ability to predict prognosis, we evaluated the IMS of all cancer samples in the TCGA database (33 cancers). Significant differences in overall survival rates were observed between the low and high IMS groups in 11 independent TCGA cancer cohorts (Fig. 5b): the BLCA, BRCA, CESC, HNSC, LUSC, OV, READ, SKCM, STAD, THCA, and UCEC cohorts. Meta-analysis showed that the IMS had obvious pancancer effects (high IMS and low IMS, hazard ratio (HR) = 0.88 (0.81, 0.94), *P* value < 0.001).

In addition, through univariate and multivariate Cox regression analysis, we found that age, stage, postoperative adjuvant chemotherapy, and IMS were independent prognostic factors for gastric cancer (Fig. 5c). To further evaluate whether IMS is related to postoperative treatment benefits, we screened stage II–IV patients in the ACRG cohort. The results showed that in the high IMS and low IMS groups, postoperative chemotherapy reduced patient recurrence and prolonged survival time (Fig. 5d). However, in the low IMS group, postoperative chemotherapy reduced the patient’s recurrence and prolonged the survival time compared with high IMS without chemotherapy. The results indicate that patients with lower IMS can benefit more from chemotherapy after surgery, and it is recommended that patients with lower IMS undergo adjuvant chemotherapy after surgery. Later, we further analyzed the influence of postoperative radiotherapy on the prognosis of gastric cancer. The results show that postoperative radiotherapy can significantly improve the prognosis of patients with high and low IMS (Fig. 5e). In the low IMS group, postoperative radiotherapy benefited more.

### IMS may be an indicator to predict the efficacy of immunotherapy for gastric cancer

**Fig. 6:**
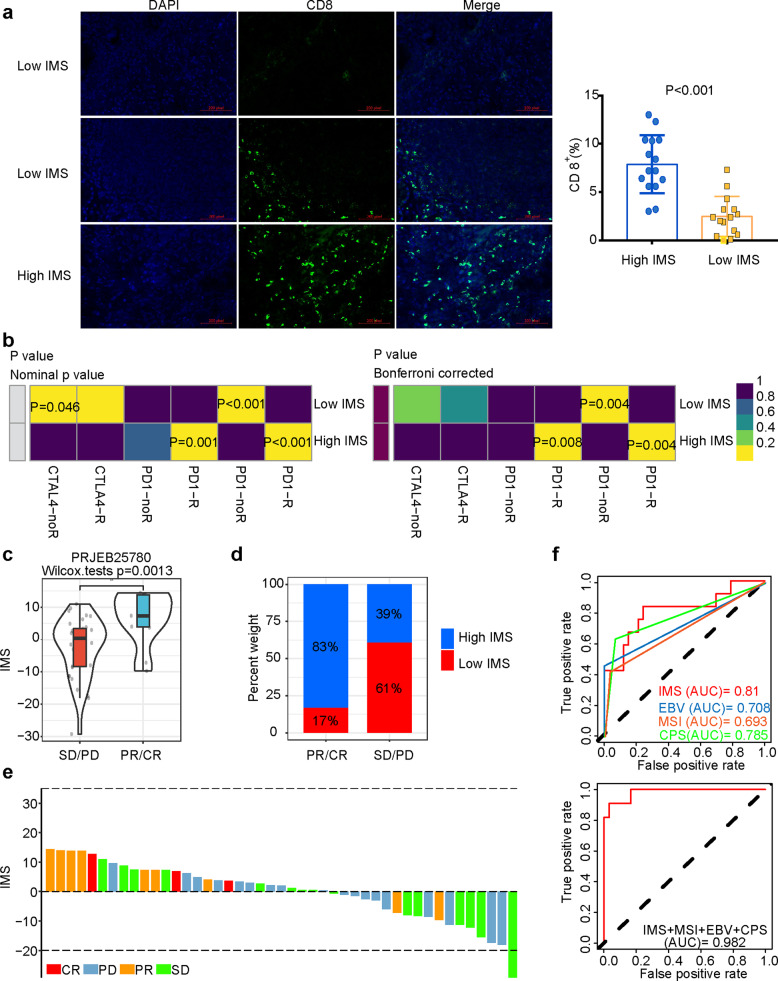
Prediction of the response to immune checkpoint inhibitor treatment. **a** Immunofluorescence staining revealed that the expression of CD8 T cell infiltration and PD-L1 protein in the high IMS group was significantly higher than that in the low IMS group (Wilcoxon test, *P* value < 0.05). **b** The tumor immune dysfunction and exclusion (TIDE) algorithm showed that the high IMS group responded to PD-1/PD-L1 inhibitor treatment (*P* value < 0.05), while the low IMS group did not respond to immune checkpoint inhibitor treatment (*P* value > 0.05). **c** The complete response (CR)/partial response (PR) group had a higher IMS than the stable disease (SD)/progressive disease (PD) group (Wilcoxon test, *P* value = 0.0013). **d** Proportion of patients responding to PD-L1 inhibitor immunotherapy: CR/PR and SD/PD: 83%/17% in the high IMS group and 39%/61% in the low IMS group (*P* value < 0.05, *χ*^2^ test). **e** Waterfall plot illustrating the IMS according to the immunotherapy response in the PRJEB25780 cohort. **f** The predictive value of IMS, MSI status, EBV status, and CPS in PD-L1 inhibitor immunotherapy patients.

To further explore the relationship between IMS and immunotherapy, we used immunofluorescence staining to analyze CD8 cell infiltration in high and low IMS. The results showed that the CD8 cells in the high IMS group significantly infiltrated the middle of the tumor tissue compared with the low IMS group (Fig. 6a), which is consistent with the transcriptome results we analyzed. The tumor immune function abnormality and exclusion (TIDE) algorithm found that the high IMS group may respond to PD-1/PD-L1 inhibitors (Fig. 6b).

We further used the PRJEB25780 cohort (PD-L1 inhibitor to treat advanced gastric cancer) to analyze whether IMS can predict immune efficacy. The results revealed that the PR/CR group had a significantly higher IMS than the SD/PD group (Fig. 6c), and 83% of the patients in the PR/CR group had high IMS (Fig. 6d). The IMS of samples with different treatment responses are shown in Fig. 6e. It was satisfactory that the area under the curve (AUC) value of IMS for predicting immunotherapy response was higher than that of MSI status, EBV status, and combined positive score (CPS) (Fig. 6f). When we combined IMS with MSI status, EBV status, and CPS, the AUC value was as high as 0.982.

### Predicting the chemosensitivity of drugs in high IMS or low IMS

**Fig. 7:**
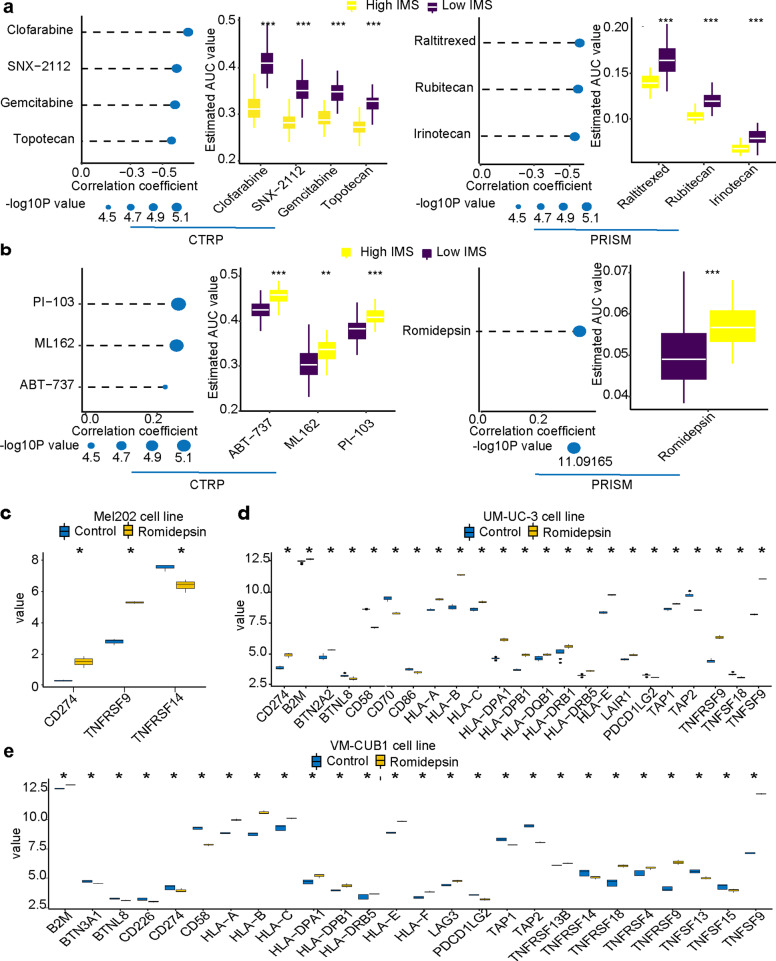
Prediction of the sensitivity to chemotherapy drugs in patients with a high and low IMS. **a** Drugs sensitive to chemotherapy in patients with a high IMS. **b** Drugs sensitive to chemotherapy in patients with a low IMS. *P < 0.05, **P < 0.01, ***P < 0.001, ****P < 0.0001. **c** Differentially expressed costimulatory and coinhibitory molecules after romidepsin treatment in the mel202 cell line. **d** Differentially expressed MHC, costimulatory, and coinhibitory molecules after romidepsin treatment in the UM-UC-3 cell line. **e** Differentially expressed MHC, costimulatory, and coinhibitory molecules after romidepsin treatment in the VM-CUB1 cell line. *P < 0.05. The center line of all boxplots represents the median value. The bottom and top of the boxes are the 25th and 75th percentiles (interquartile range). The whiskers encompass 1.5 times the interquartile range.

Immunotherapy is an important discovery in cancer therapy, but chemotherapy has always been an important strategy for postoperative treatment. Therefore, we used the CTRP and PRISM databases to predict potential therapeutic drugs for gastric cancer. The results showed that clofarabine, SNX-2112, gemcitabine, topotecan, raltitrexed, rubitecan, and irinotecan were more suitable for patients with high IMS (Fig. 7a). For patients with low IMS, ABT-737, ML162, PI-103, and romidepsin may be effective for treatment (Fig. 7b). Among them, we found that the AUC value of romidepsin was the lowest among these 11 chemotherapy drugs, which implies that romidepsin may have good treatment sensitivity in high IMS and low IMS.

With this discovery, we searched the dataset of romidepsin treatment of cancer through the Gene Expression Omnibus (GEO) database. In GSE155452 uveal melanoma cell line (mel202), after romidepsin intervention, the expression of CD274 was significantly increased, and the expression of TNFRSF9 and TNFRSF14 was significantly different (Fig. 7c). In GSE70120, bladder transitional cell carcinoma (UM-UC-3) also showed significantly increased expression of CD274 and significantly high expression of MHC molecules and coinhibitory and costimulatory molecules (Fig. 7d). In the VM-CUB1 cell line, however, although the expression of CD274 was significantly reduced, significantly high expression of MHC molecules and coinhibitory and costimulatory molecules was still found (Fig. 7e). This result suggests that after romidepsin treatment, tumor cells are more likely to be exposed to immune monitoring. Immune cells may recognize and kill tumor cells faster, but there is also immunosuppression (increased expression of CD274).

**Fig. 8:**
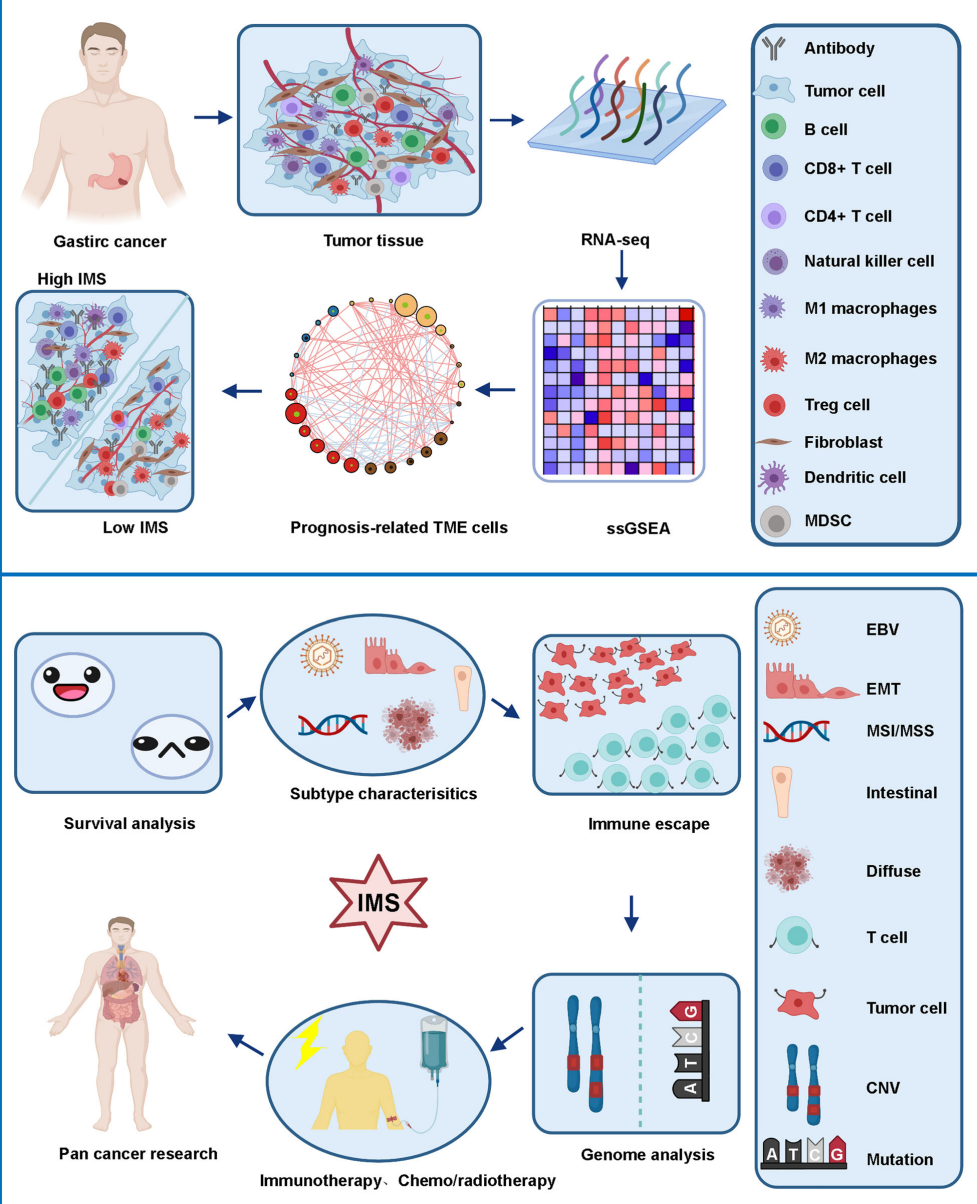
The illustrations for this study. Identify gastric cancer immune subtype characteristics based on IMS and guide prognosis and treatment strategies.

In summary, our findings strongly suggest that IMS was significantly associated with tumor phenotype and further elucidate the potential mechanisms and therapeutic strategies between the high and low IMS groups. IMS is a strong favorable marker for gastric cancer. The illustrations for this study are shown in Fig. 8.

## Discussion

Immunotherapy has shown strong antitumor activity in the treatment of solid tumors such as melanoma, nonsmall-cell lung cancer, and prostate cancer^29–31^. Although the success of immunotherapy is exciting, immunotherapy still faces many challenges^32^. Through in-depth exploration, researchers have realized that the TME is complex and diverse in terms of immune status, and different aspects of the TME have been shown to have important impacts on prognosis and the efficacy of immunotherapy^33,34^. In this study, gastric cancer patients were stratified according to characteristics of the TME, and these results will deepen the understanding of the effects of the TME on antitumor and immune responses and will guide more effective immunotherapy strategies.

In this study, we constructed the IMS for each sample based on 27 types of cells in each independent gastric cancer cohort. Patients with a high IMS had better overall survival rates than those with a low IMS. We also explored whether the IMS had a strong ability to assess prognosis. To do so, we extended our analysis to include 33 cancers from the TCGA database. A high IMS was found to be a potential protective factor (HR < 1) in 19 cancers (Fig. 5b). Significant differences in overall survival were observed in 11 independent cohorts: the BLCA, BRCA, CESC, HNSC, LUSC, OV, READ, SKCM, STAD, THCA, and UCEC cohorts. Although the results of the subgroup analysis were heterogeneous, the meta-analysis revealed that the IMS had obvious pancancer effects (HR = 0.81 (0.76, 0.87), *P* value < 0.01). It may be a powerful marker for predicting the prognosis of cancer.

We further analyzed the characteristics of the high and low IMS groups in gastric cancer. Increasing evidence suggests that solid tumors can be divided into an immune inflammatory type (hot tumor), characterized by adaptive immune activation, and an immune exclusion type (cold tumor), with innate immunity and interstitial activation^15,21,35^. Our research revealed activation of adaptive immunity in the high IMS group and innate immunity and interstitial activation in the low IMS group (Fig. 2c). Although the low IMS group also showed immune cell infiltration, the infiltrating immune cells were located only around cell nests surrounded by matrix, suggesting that the cells could not enter the parenchyma, leading to the failure of antitumor immunity^36^. We also verified our findings with the result (Fig. 6a). Some studies have found that patients with the MSI and EBV subtypes of disease are sensitive to immune checkpoint inhibitor therapy^37–39^. EBV-positive tumors have low TMB but strong immune infiltration^40^. Matrix activation in the EMT and gastric cancer subtypes has been identified as the main factor leading to the failure of checkpoint inhibitor therapy^41^. Our study found that the IMS was significantly increased in the EBV subtype and MSI subtype, while the IMS was lowest in the EMT subtype and gastric cancer subtype. Moreover, the EMT and TGF-β signaling pathways were activated in patients with a low IMS, and extensive immune pathways and DNA damage response pathways were activated in patients with a high IMS. These results are consistent with the results of previous studies. These results further show that the IMS is a powerful and robust tool for stratifying patients with gastric cancer and further determining the immunophenotype of gastric cancers.

The immune escape mechanisms of tumors play an important role in tumor treatment, especially immunotherapy. We further explored the immune escape mechanisms in the high and low IMS groups. The expression of chemokines, ILs, and interferon was significantly lower in the low IMS group than in the high IMS group (Supplementary Fig. 4a), and the expression of these molecules was significantly related to tumor extrinsic immune escape mechanisms^28^. The immunogenicity of tumors and the expression of checkpoint molecules are the most important factors related to intrinsic escape^42^. Blocking the PD-1/PD-L1 pathway can induce immune cells to kill tumors and has been shown to provide a long-lasting response in a variety of cancers^43–45^, including gastric cancer. However, it has been found that this method is only applicable in a small number of patients, and multiple studies have found that PD-L1 expression, MSI status, and TMB are biomarkers for predicting immune benefits^37,46^. Our results found that TMB and MSI status were not significantly related to the expression of most immune checkpoint molecules. However, what is very surprising is that the IMS was significantly related to the expression of the immune checkpoint molecules TMB and MSI (Fig. 3c). The high IMS group showed significantly elevated expression of MHC class I molecules, and research reports have confirmed that tumors can escape T cell attack by suppressing MHC class I molecule expression^47^. These results further prove that the IMS we established may be an effective targeted predictive biomarker. To further understand the difference between the high and low IMS groups, we analyzed the whole-exome sequencing data of gastric cancer samples categorized according to the IMS. We found that patients with a low IMS had significantly higher copy number gain/loss frequencies than patients with a high IMS. This result is very similar to previous reports^48^. We explored the gene mutations in the high and low IMS groups. The results showed that the frequency of gene mutations in the high IMS group was significantly higher than that in the low IMS group. This difference may be one of the reasons for the increased TMB in patients with a high IMS.

To explore the relationship between IMS and immunotherapy, we used the TIDE algorithm to predict the response of high IMS and low IMS to immunoassay inhibitor (anti-CTLA4 and anti-PD-1/anti-PD-L1) treatment. The results showed that high IMS responded to anti-PD-1/anti-PD-L1 treatment in gastric cancer, while low IMS did not (Fig. 6b). In addition, we used the PRJEB25780 cohort (PD-L1 inhibitor) to evaluate some indicators for the evaluation of immune efficacy. Our research also found that MSI status (AUC = 0.693), EBV infection status (AUC = 0.708), and CPS (AUC = 0.785) have good predictive power, but to our satisfaction, IMS has the highest AUC value (AUC = 0.81). And when we combined these indicators to predict immunotherapy response, we found that the predictive ability was greatly improved (AUC = 0.982, Fig. 6f). This also further suggests that IMS is a potential indicator for predicting the response of gastric cancer immunotherapy.

To further explore therapies for patients with low IMS, we predicted that romidepsin may be suitable for patients with low IMS through the PRISM database (Fig. 7b). Related studies have also found that romidepsin treatment may increase tumor immune infiltration, but it also promotes the expression of PD-1/PD-L1. We further analyzed the results of three cancer cell lines (mel202, UM-UC-3, and VM-CUB1) after romidepsin intervention (Fig. 7). Interestingly, we found that after romidepsin intervention, the expression of MHC molecules, cosuppressor genes, and costimulatory genes increased significantly. This suggests that this low IMS patient may change from an EMT subtype (cold tumor) to an immune-infiltrating subtype (hot tumor) after romidepsin treatment. This provides very exciting news for the treatment strategy of patients with low IMS. Patients with low IMS may benefit more from romidepsin combined with immunotherapy.

There are some limitations to this study. First, we analyzed the heterogeneity of the TME and estimated the IMS of each sample, but we did not consider the heterogeneity within the tumor, which is also an important factor affecting prognosis and treatment. Second, classifying the gastric cancer samples according to only the median IMS value was not a robust way to categorize the samples, but as correlation analysis was used in our research, this problem was somewhat lessened; however, more effective methods may be needed to explore the best cutoff value. Third, we performed genomic analysis, which does not reflect the cause of the event, and more in-depth basic research may be required in the future.

In summary, this work demonstrates the potential molecular mechanisms affecting the differences between high- and low-IMS samples. This study helps us understand the distribution of immune infiltration and immune escape mechanisms in gastric cancer. The IMS can be used to stratify patients and identify those who will benefit more from immunotherapy and to uncover new strategies for cancer treatment.

## Methods

### Data acquisition and preprocessing

The gene expression profiles and corresponding clinical information of patients with gastric cancer were obtained from the GEO and TCGA databases. The postoperative chemotherapy and radiotherapy dataset for gastric cancer was obtained from the MD Anderson Cancer Center cohort (GSE28541). The PD-L1 treatment cohort for gastric cancer was obtained from the European Nucleotide Archive database (PRJEB25780). The transcriptome data of the cell line before and after romidepsin treatment were obtained from GSE155452 and GSE70120. These samples were not treated before surgery.

A total of 1422 gastric cancer samples from six cohorts (GSE84437, GSE34942, GSE15459, GSE57303, ACRG cohort, and TCGA-STAD (stomach adenocarcinoma)) were included in the study. These patients were not treated before surgery. The “CEL” file for the microarray data from Affymetrix^®^ was downloaded from GEO (https://www.ncbi.nlm.nih.gov/geo/). The data were standardized using the robust multiarray averaging method with the “affy” and “simpleaffy” packages^49^. For microarray data from other platforms, the normalized matrix files were downloaded from GEO. For the cohorts from TCGA, RNA sequencing data (in fragments per kilobase per million (FPKM)) were downloaded from the Genomic Data Commons (GDC) data portal (https://portal.gdc.cancer.gov/). Then, the FPKM values were transformed into transcripts per kilobase million (TPM) values.

### Gene set enrichment analysis and functional annotation

Gene set enrichment analysis (GSEA) of the gastric cancer cohorts was performed with GSEA software (version 4.1.0). ssGSEA was used to calculate the standardized enrichment score via the GSVA package^50^. ssGSEA ranks the gene expression values of a sample and uses the empirical cumulative distribution function of the genes in the signature and the rest of the genes to generate enrichment scores. Fifty-one TME cell signatures were collected via a literature search, and they were considered reliable^26,51–53^. The gene set file “c2.cp.kegg.v6.2” was downloaded from the Molecular Signatures Database (https://www.gsea-msigdb.org/gsea/index.jsp). The immune checkpoint signature, antigen processing machinery signature, CD8 T cell effector signature, WNT target signature, EMT signature, angiogenesis characteristics signature, Pan-F TBRS signature, and repair of DNA damage signature were obtained from previous studies^27,51,54^. The R package clusterProfiler was used for functional annotation of genes^55^.

### Calculation of the immune microenvironment score (IMS) for gastric cancer

The NES of each immune cell was obtained by ssGSEA for each gastric cancer sample. Univariate Cox regression analysis was used to evaluate the relationship between the NES and overall survival in each gastric cancer cohort. Then, a meta-analysis was used to estimate the overall HR and *P* value with the R package meta. Only immune cell signatures with an overall *P* value less than 0.05 in the meta-analysis were included in the study^56^.

Finally, the IMS for each sample in each cohort was defined as follows:

1 $${\rm{IMS}} = \mathop {\sum }\limits_{i = 1}^n {\rm{NES}} - \mathop {\sum }\limits_{j = 1}^m {\rm{NES}}$$

where NES*i* is the NES of immune cell signatures with an HR less than 1 in the overall cohort and NES*j* is the NES of immune cell signatures with an HR more than 1 in the overall cohort.

### Collection of immune-related data

Several bioinformatics immune parameters were considered, such as TMB, HRD, ITH, aneuploidy score, CTA expression, LOH, TAI, and LST. The results for these immune parameters for gastric cancer patients were collected from GDC (https://gdc.cancer.gov/about-data).

### Comparison of genomic alterations in different gastric cancer subtypes

Gastric cancer mutation data (VarScan2) were downloaded from GDC. Genes with mutation frequencies less than 2.5% were excluded from the analysis. Gastric cancer SCNV data were collected from GDC. The GISTIC score and gene copy number amplification and deletion data for each sample were analyzed by GISTIC 2.0 software. The FGA value of each gastric cancer sample was determined^57^. The position of the gene on the chromosome was visualized with the R package RCircos.

### RNA sequencing analysis of gastric cancer samples

A total of 36 fresh gastric cancer samples were collected from Peking University People’s Hospital. All patients had signed an informed consent form, and the research protocol was approved by the Ethics Committee of Peking University People’s Hospital. These patients were not treated before surgery. Two tumor tissues were collected immediately after each specimen was isolated: one was stored in liquid nitrogen, and the other was fixed in formalin. According to the manufacturer’s instructions, an RNA extraction kit (ER501-01, TransGen Biotech, Beijing, China) was used to extract sample RNA from samples stored in liquid nitrogen. A NanoDrop was used to detect RNA purity, and an Agilent 2100 bioanalyzer (Thermo Fisher Scientific, USA) was used to detect RNA concentration and integrity. Next, an mRNA library was constructed, the RNA was fragmented into small fragments, and cDNA was combined. After incubating the cDNA fragment with A-tailing mix and RNA Adaptor Index for end repair, it was further amplified by PCR. Then, qualified double-stranded PCR products were used to construct the final library. A total of 30 qualified samples were further sequenced on the BGISEQ-500 platform (Beijing, China). TPM was used to calculate the expression level of genes.

### Immunofluorescence staining

The gastric cancer tissue block was embedded in paraffin and continuously cut into 3 µm sections and placed on a glass slide. The slices were baked at 72 °C for 1 h, dewaxed with xylene, and dehydrated with gradient alcohol. After rinsing five times with phosphate-buffered saline (PBS), 2 min each time (1‰ Tween 20 is added to PBS), then after high-pressure repair, rinse again with PBS five times. The processed sections were immersed in a 3% hydrogen peroxide solution, incubated at room temperature for 10 min, and washed with distilled water and PBS. Next, CD8 mouse-derived primary antibody (Santa Cruz, sc-70794, USA; 1:100) was added to the slices in equal proportions, incubated at 37 °C for 1 h, and rinsed with PBS three times for 5 min each time. Alexa Fluor 488-labeled goat anti-mouse IgG (Jackson, 115-545-003, USA; 1:1000) was added in equal proportions of secondary antibody mixture. After incubating at 37 °C for 25 min, the cells were rinsed with PBS three times for 5 min each time, allowed to dry and mounted with 4’,6-diamidino-2-phenylindole, dihydrochloride (Invitrogen, S36942, USA). An AXIO Scan. Z1 scanner was used to scan. Finally, the percentage of positive cells was counted, and the results were confirmed by two pathologists.

### Prediction of immunotherapy response and chemotherapeutic drug sensitivity

The TIDE algorithm (http://tide.dfci.harvard.edu/) and submap algorithm (https://cloud.genepattern.org/gp) were used to predict the clinical response of patients with high- or low-IMS subtypes to immune checkpoint inhibitors^58^. The CTRP2.0 database and PRISM database were used to predict the sensitivity of high- or low-IMS-subtype chemotherapeutics by referring to the method of Yang et al. The CTRP2.0 database contains sensitivity data of 481 compounds, and the PRISM database contains sensitivity data of 1448 compounds. According to PRISM and CTRP2.0, which contains data on the drug sensitivity AUC value, these two data sets use the AUC as a measure of drug sensitivity, and a lower AUC value indicates increased sensitivity to treatment^59^. We used the cell line expression profile of the CCLE database as the training set for drug sensitivity prediction and TCGA-STAD as the test set.

### Statistical analysis

Univariate Cox regression analysis was used to evaluate the relationship between features of interest and overall survival. The limma package in R was used to determine the differentially expressed signaling pathways in the gastric cancer cohorts. The ggplot2 package and ComplexHeatmap package were used to draw heatmaps and other maps. The R package forestplot was used to draw forest plots, and the R package meta was used for meta-analysis. The correlation coefficient between immune cell inflammation was determined by Spearman’s and distance correlation. The Wilcoxon rank sum test was used to analyze the difference between two groups. One-way ANOVA and the Kruskal–Wallis test were used to compare differences between three or more groups. Correlation matrices were created with Pearson’s or Spearman’s correlation.

The overall survival curve was estimated by the Kaplan–Meier method, and the difference between survival distributions was evaluated by the two-sided log-rank test implemented in the R package survival. The R package survminer was used to draw the Kaplan–Meier survival curve. The specificity and sensitivity of the IMS were assessed through receiver operating characteristic curves, and the AUC was quantified using the pROC R package.

All statistical *P* values were two-sided, and *P* < 0.05 was considered statistically significant. All analyses were performed with R software (version 4.0.2).

### Reporting summary

Further information on research design is available in the Nature Research Reporting Summary linked to this article.

## Supplementary information

Supplementary Information

Supplementary Data 1

Supplementary Data 2

Supplementary Data 3

Supplementary Data 4

Supplementary Data 5

Supplementary Data 6

Reporting Summary

## Data Availability

The gastric cancer data involved in this study are from TCGA (https://www.cancer.gov/) and GEO (https://www.ncbi.nlm.nih.gov/geo/) databases. The sequencing data of this study were stored in the GEO database (GSE180887). The aggregate data are available from the corresponding author on reasonable request.

## Code availability

No previously unreported computer code or algorithm was used. Data analysis was performed using standard tools, as described.
