# Supplementary material for: Immune cell infiltration signatures identified molecular subtypes and underlying mechanisms in gastric cancer
Source: NPJ Genom Med. 2021 Oct 11;6:83. doi: 10.1038/s41525-021-00249-x (PMC8505616; doi:10.1038/s41525-021-00249-x)
Supplement: Supplementary file 1 — Supplementary Information [file 41525_2021_249_MOESM1_ESM.pdf]

Immune cell infiltration signatures identified molecular subtypes and underlying mechanisms in gastric cancer

Supplementary Data 1: Basic information of the data set used for gastric cancer analysis in this study

This file is supplied as a separate excel file (Supplementary Data 1.xlsx)

Supplementary Data 2: Univariate Cox regression analysis of immune cell signatures in each gastric cancer cohort

This file is supplied as a separate excel file (Supplementary Data 2.xlsx)

Supplementary Data 3: Significantly different immune cell signatures revealed by the meta-analysis in gastric cancer

This file is supplied as a separate excel file (Supplementary Data 3.xlsx)

Supplementary Data 4：Gene set of if immune cell types with significant prognostic differences

This file is supplied as a separate excel file (Supplementary Data 4.xlsx)

Supplementary Data 5: The relationship between the IMS and gastric cancer subtypes

This file is supplied as a separate excel file (Supplementary Data 5.xlsx)

Supplementary Data 6: The correlation between gene mutation and the IMS

This file is supplied as a separate excel file (Supplementary Data 6.xlsx)

Supplementary Figures

Supplementary Figure 1 Overview of the study design

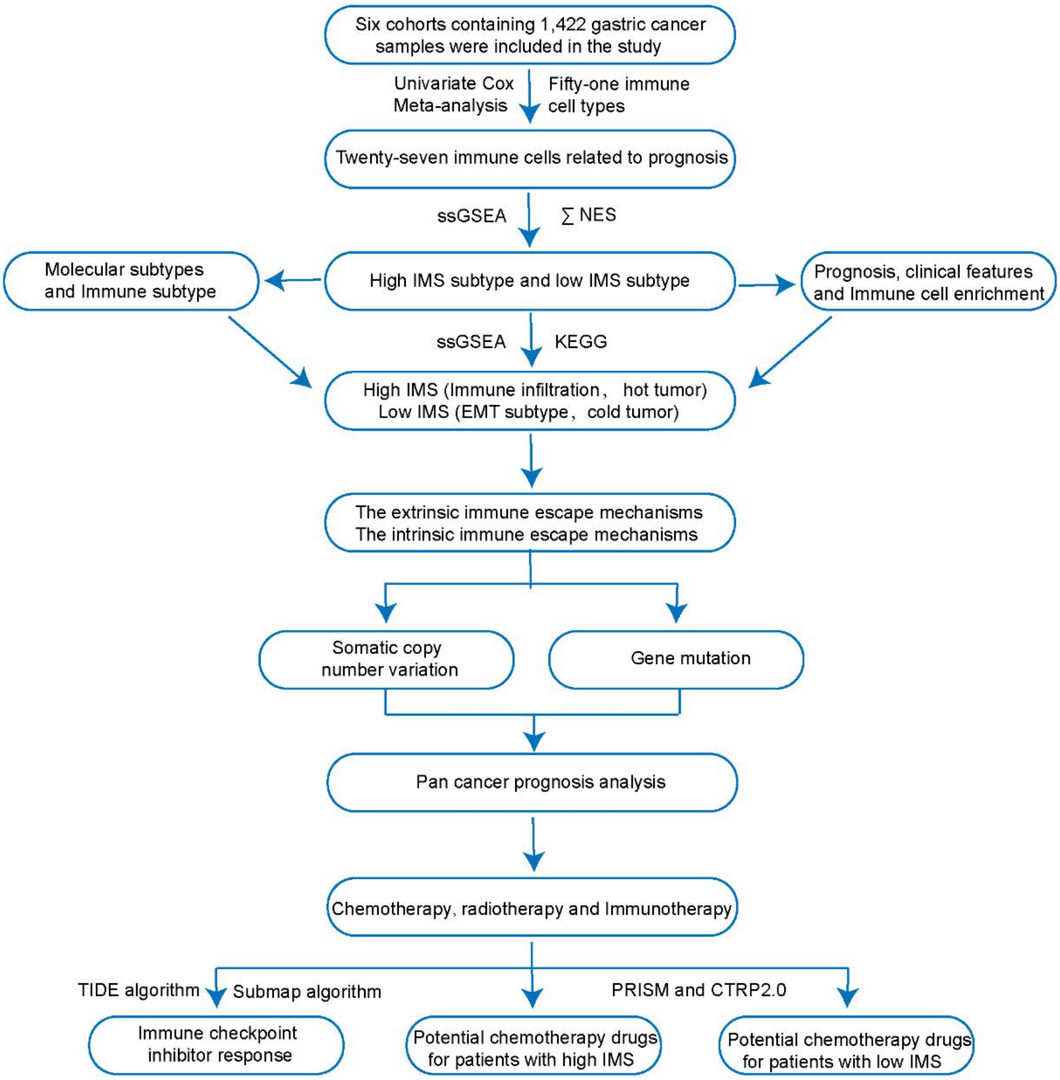

Supplementary Figure 2 Landscape of prognosis-related immune cells

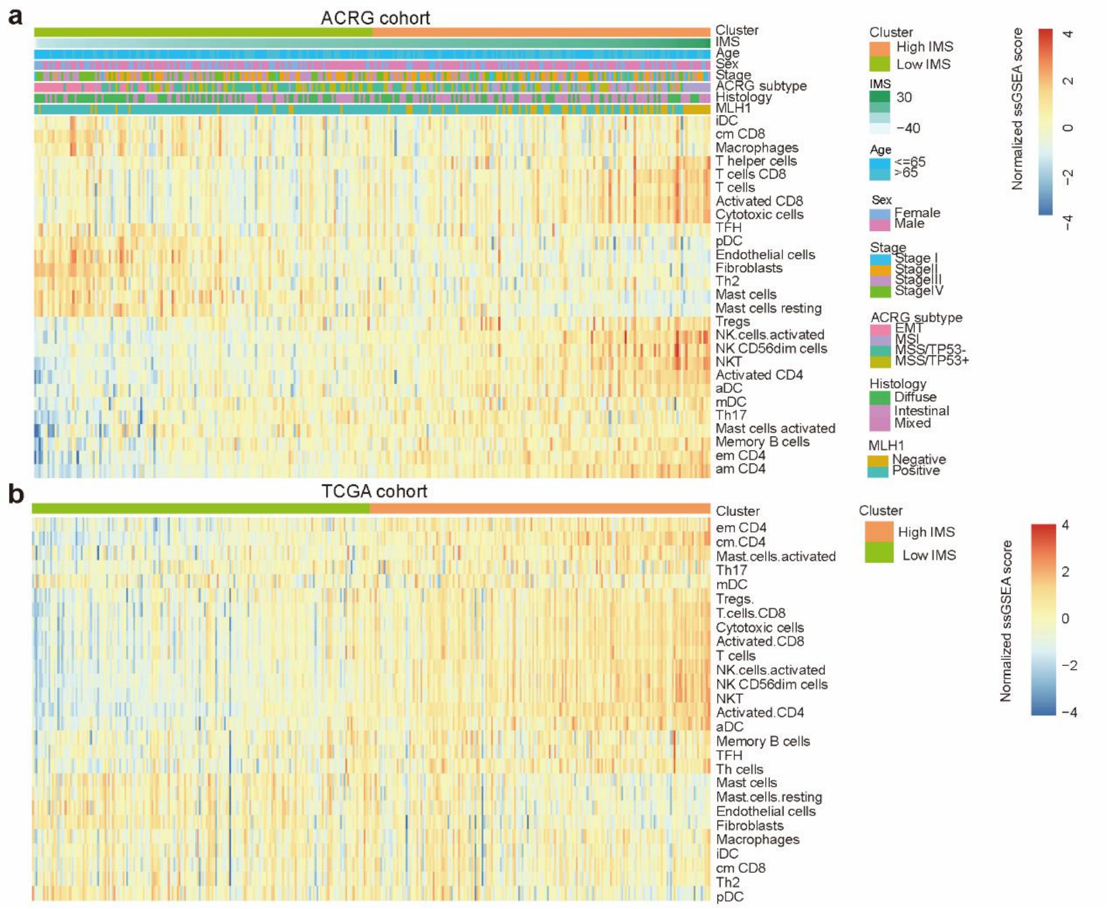

(a) Heatmap showing the expression of 27 immune cell types with differential prognostic significance between the high and low IMS groups. The samples were sorted according to the IMS value from small to large (ACRG cohort).

(b) Heatmap showing the expression of 27 immune cell types with differential prognostic significance in the TCGA cohort.

Supplementary Figure 3 The relationships between clinical features and

the IMS

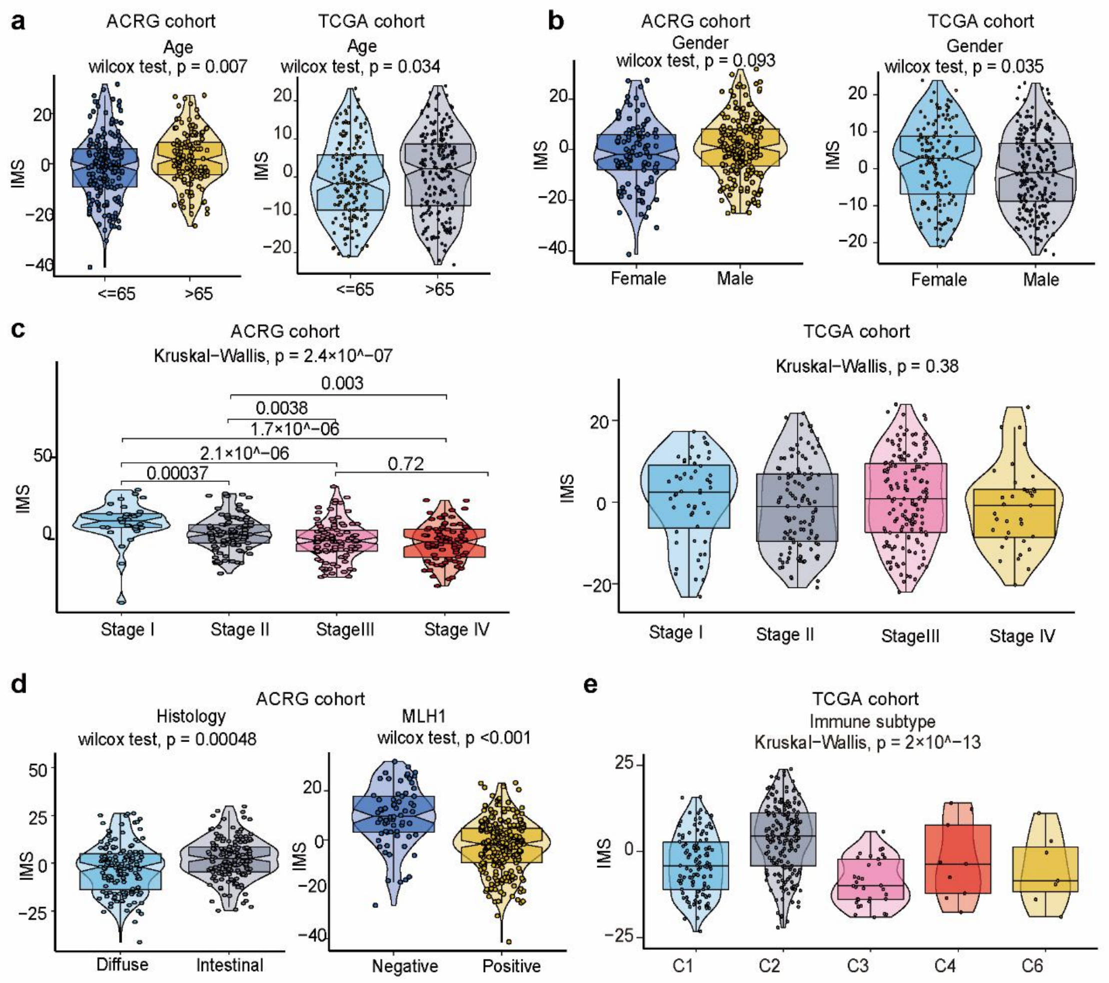

(a) Differences in the IMS among patients of different ages (Wilcoxon test). (b) Differences in the IMS among male and female patients (Wilcoxon test). (c) Differences in the IMS according to different AJCC stages (Kruskal-Wallis). (d) Differences in the IMS according to different Lauren subtypes and MLH1 statuses (Wilcoxon test). (e) Differences in the IMS according to different subtypes (immune subtypes, Kruskal-Wallis). The center line of all boxplots represents the median value. The bottom and top of the boxes are the 25th and 75th percentiles (interquartile range). The whiskers encompass 1.5 times the interquartile range.

Supplementary Figure 4 Correlation between the IMS and potential

extrinsic immune escape mechanisms in the TCGA gastric cancer cohort

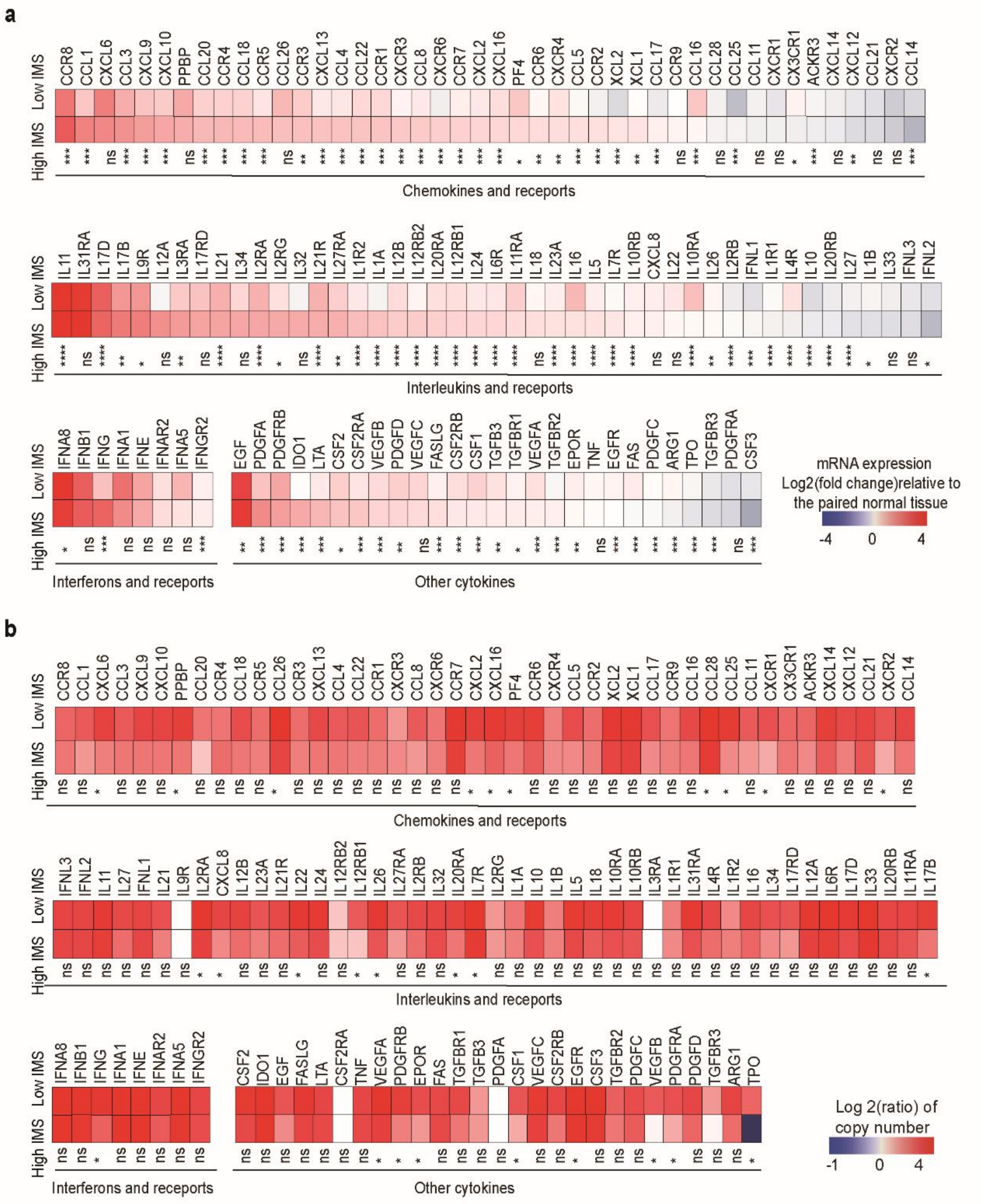

(a) Comparison of the log2-fold changes in chemokines, interleukins, interferon and other important cytokines and their receptors at the tumor sites relative to those in paired normal tissue. (b) Comparison of the log2 ratio of the copy number values of chemokines, interleukins, interferon and other important cytokines and their receptors in the high and low IMS groups. (* p<0.05, ** p<0.01 *** p<0.001, **** p<0.0001, ns P-value > 0.05)

Supplementary Figure 5 The prognostic value of the IMS in six independent gastric cancer cohorts

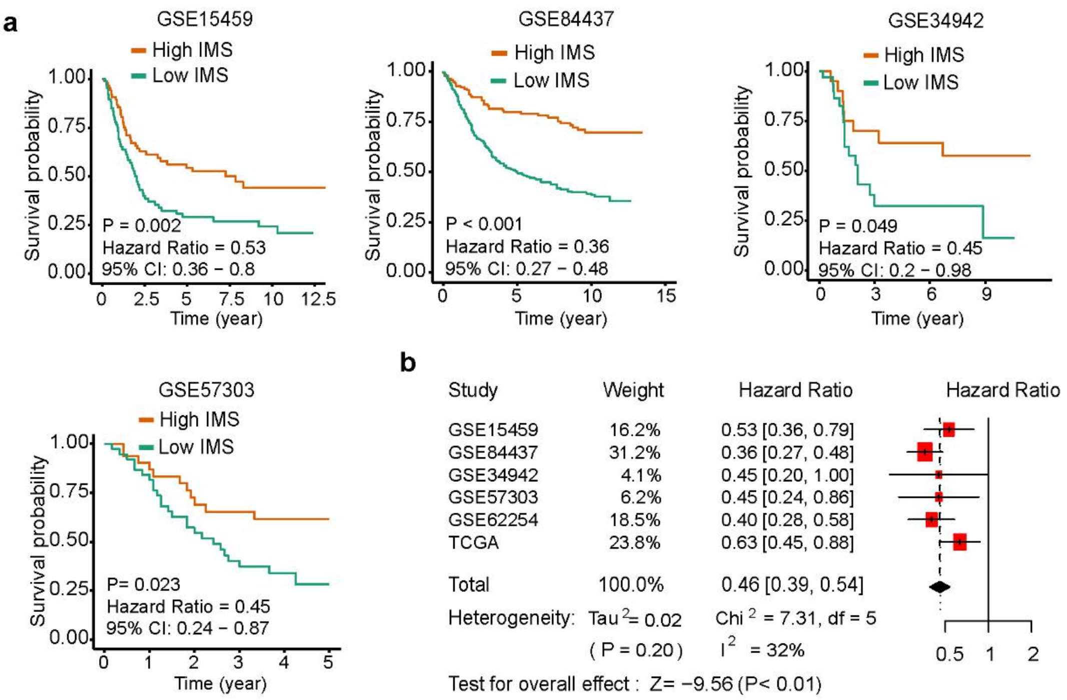

(a) The Kaplan-Meier method was used to assess the overall prognostic significance of the IMS in gastric cancer. (b) A fixed-effect model was used to evaluate the overall prognostic significance of the IMS by meta-analysis.
